# Body mass index and physical activity in seven-year-old children whose mothers exercised during pregnancy: follow-up of a multicentre randomised controlled trial

**DOI:** 10.1186/s12887-021-02952-1

**Published:** 2021-11-08

**Authors:** Karen Alterhaug Bjøntegaard, Signe Nilssen Stafne, Siv Mørkved, Kjell Åsmund Salvesen, Kari Anne I. Evensen

**Affiliations:** 1 Faculty of Medicine and Health Sciences, Norwegian University of Science and Technology (NTNU), Trondheim, Norway; 2 Department of Public Health and Nursing, Norwegian University of Science and Technology (NTNU), Trondheim, Norway; 3 Clinic of Clinical Services, St. Olavs Hospital, Trondheim University Hospital, Trondheim, Norway; 4 Department of Clinical and Molecular Medicine, Norwegian University of Science and Technology (NTNU), Trondheim, Norway; 5 Department of Obstetrics and Gynecology, St. Olavs Hospital, Trondheim University Hospital, Trondheim, Norway; 6 Department of Physiotherapy, Oslo Metropolitan University, Oslo, Norway

**Keywords:** Child, Body mass index, Exercise during pregnancy, Follow-up, Physical activity, Randomised controlled trial

## Abstract

**Background:**

There are limited data on long-term outcomes of children whose mothers have followed exercise interventions during pregnancy. The aim of this paper was to investigate whether regular moderate intensity exercise during pregnancy affected the children’s body mass index (BMI) and physical activity (PA) at 7 years of age, and determine the relationship between children’s and mothers’ BMI and PA.

**Methods:**

This was a follow-up of a multicentre randomised controlled trial, carried out at St. Olavs Hospital, Trondheim University Hospital, and Stavanger University Hospital, Norway (2007–2009 and 2014–2016). Women were randomised to follow a 12-week structured exercise protocol or standard antenatal care during pregnancy. At the 7-year follow-up, parents reported their child’s height, weight, and PA. The mothers also reported their own weight and PA. Main outcome variables were BMI, frequency and duration of moderate to vigorous PA (MVPA), and intensity of PA.

**Results:**

A total of 855 women were randomised to exercise (*n* = 429) or standard antenatal care (*n* = 426) during pregnancy. At follow-up, 164 (38.2%) children and mothers in the intervention group and 117 (27.5%) in the control group participated. We found no group differences in the children’s iso-BMI or PA. Findings were similar when we performed stratified analyses by sex, except boys in the control group spent more time on electrical devices than boys in the intervention group. Subgroup analyses of children of mothers who adhered to the exercise protocol and sensitivity analyses excluding children born preterm, children admitted to the neonatal intensive care unit, and children with diseases or health problems at the 7-year follow-up, did not change the results. Children’s BMI, weekly leisure time MVPA and intensity of PA correlated with mothers’ BMI, daily exercise, and intensity of exercise.

**Conclusions:**

Regular moderate intensity exercise during pregnancy did not affect BMI or PA of the children at 7 years. Good maternal health should be encouraged as it may influence the health of the next generation.

**Trial registration:**

The initial RCT study was registered in ClinicalTrials.govNCT00476567.

**Supplementary Information:**

The online version contains supplementary material available at 10.1186/s12887-021-02952-1.

## Background

Pregnancy is an important period with respect to childhood development and future health [1–3]. Current guidelines for physical activity (PA) during pregnancy recommend 30–60 min of moderate intensity PA at least 3–4 days per week [4], or at least 150 min of weekly moderate intensity exercise [5, 6]. Exercise during pregnancy has potential to influence the intrauterine environment through modulation of epigenetics [7], placental blood flow, nutrient partitioning, and oxygen delivery to the foetus [8]. There is growing evidence that exercise during pregnancy causes no harm to the children and benefits have been documented for cardiovascular health [9], nervous system development [10–15], and academic performance [16].

However, there are limited data on long-term outcomes of children whose mothers have followed exercise interventions during pregnancy. Even though there are clear benefits of PA for all [17], none has examined PA as an outcome in children born to mothers who exercised during pregnancy. Case-control studies have shown that children of exercising women weighed less and had less body fat than children of control group women at birth [18] and at 5 years [19], but not at 1 year of age [20]. A randomised controlled trial (RCT) follow-up study found that children born to mothers in the exercise group weighed less than control group children at birth, but had more body fat at 7 years [21]. This indicates potential adverse outcomes in mid-childhood, raising questions about the guidelines for exercise during pregnancy [4].

Not only prenatal, but also postpartum, maternal health may influence the child’s future health. Since pregnancy may be a “golden opportunity” for sustainable lifestyle changes, it is possible that PA during pregnancy may have long-term effects also through increased PA during childhood years. Two prospective cohort studies of mother-child pairs showed that a healthy lifestyle of the mother, including regular exercise and a healthy body mass index (BMI), during their offspring’s childhood and adolescence substantially reduced the risk of obesity in the offspring [22]. Furthermore, parental adiposity is associated with offspring birth weight and risk of later child obesity [23, 24].

We have previously reported that exercise in pregnancy is safe [25], and does not have adverse effects on the child’s neurodevelopment at 18 months [10] or 7 years of age [11]. In the current study, our primary aim was to investigate whether an exercise intervention during pregnancy affected BMI and PA of the children at 7 years of age. Furthermore, we wanted to determine the relationship between the children’s and the mothers’ BMI and PA. We hypothesised that there would be no group differences in BMI or PA, but that the children’s BMI and PA would correlate with that of the mothers.

## Methods

### Study design

This is a follow-up study of a Norwegian multicentre RCT that investigated whether regular exercise during pregnancy could prevent gestational diabetes [25]. Pregnant women booking appointments for routine ultrasound scans at St. Olavs Hospital, Trondheim University Hospital, and Stavanger University Hospital were invited to participate.

In total, 875 of approximately 12,000 women accepted participation during the inclusion period from April 2007 to June 2009. Of these, 855 women in week 18–22 of pregnancy were randomly assigned to receiving a 12-week standard exercise program (intervention group) or standard antenatal care (control group). Inclusion criteria were Caucasian women aged 18 years or older with a singleton live foetus. We included only Caucasian women to ensure an ethnically homogenous sample as ethnicity is a risk factor for gestational diabetes [26, 27]. The age limit was set due to age of majority in Norway, and singleton live foetus to avoid pregnancy complications. Exclusion criteria were high-risk pregnancies, diseases that could interfere with participation and women living too far away (> 30 min’ drive) from the hospitals. Randomisation in blocks of 30 was performed by a web based computerised procedure at Unit for Applied Clinical Research, Norwegian University of Technology and Science. Because of the nature of the study, physiotherapists leading group sessions and study participants were not blinded. The women were examined at baseline (week 18–22 of pregnancy), at end of intervention (week 32–36 of pregnancy) and 3 months after delivery [28]. Pregnancy outcome and newborn data were registered at time of delivery [25]. The children were clinically assessed at 18 months of age [10] and by parent-report at 7 years of age [11].

Data for the 7-year follow-up were collected electronically using the CHECKWARE software (CheckWare AS, Trondheim, Norway) from October 2014 to December 2016. Parents of all 855 children received the questionnaire in the autumn term of their child’s second year of primary school.

### Intervention group

Women in the intervention group received a standardised exercise program including aerobic activity of moderate intensity, strength training and balance exercises as recommended by the American College of Obstetricians and Gynecologists and the Norwegian Directorate of Health [6, 29]. Training sessions of 60 min instructed by a physiotherapist were offered once per week over a period of 12 weeks (between week 20 and 36 of pregnancy). In addition, the women were encouraged to follow a written 45-min home exercise program at least twice per week, including 30 min of endurance training and 15 min of strength and balance exercises. Exercises were shown in text and pictures in a booklet and instructed at group sessions. Adherence to the protocol was defined as exercising 3 days per week or more at moderate to high intensity and participating in at least one group session per week [25]. Moderate intensity was defined as getting sweaty and a little out of breath during exercise. Performing the exercise programme was strongly emphasised and recorded in the women’s personal training diaries and through reports from physiotherapists leading the training groups. In addition, PA was recorded in a questionnaire for both groups.

### Control group

Women in the control group received standard antenatal care in Norway and the customary information given by their midwife or general practitioner. They were not discouraged from exercising on their own [25].

### Outcome variables

At the 7-year follow-up, we used questions regarding height, weight, diseases and health problems from a questionnaire developed by the National Institute of Health for the Norwegian Mother and Child Cohort (MoBa) study [30, 31]. The children’s height was reported in cm and their weight in kg. BMI was calculated as weight in kilogram divided by the square value of height in meters (kg/m^2^). We calculated the children’s iso-BMI, i.e. BMI adjusted for sex and age, by using a weight calculator [32]. We used iso-BMI as a continuous variable and as a dichotomised variable with 25 kg/m^2^ as a cut off for overweight [32, 33]. Parents reported whether their child had any of the following diseases or health problems; rheumatoid arthritis, cancer, diabetes, cerebral palsy, attention-deficit hyperactivity disorder, coeliac disease, bone fractures, epilepsy, mental retardation, autistic traits, Asperger’s syndrome, chronic fatigue syndrome/myalgic encephalomyelitis, tonsillectomy, ear drainage or other conditions or congenital diseases [31].

The PA questions were the same as used in a Norwegian cohort study in the county of Telemark [34, 35]. Two of the questions were equal to questions from the WHO Health Behaviour in School-aged Children questionnaire [36], also used in the large Norwegian Young-HUNT study [37, 38]. In order to assess whether the child met the recommendation from the Norwegian Directorate of Health [6] to perform 1 h or more of daily moderate to vigorous PA (MVPA), parents reported total (including school, after-school program and leisure time) daily MVPA for their child as 1) Less than 1 h or 2) 1 h or more [34]. Frequency of leisure time MVPA, i.e. PA performed outside school and after-school program where the child was out of breath or sweaty, was reported as number of times per week (never/once a month or less/once a week/2 to 3 times a week/4 to 6 times a week/every day). Weekly leisure time MVPA was reported as approximate hours per week (none/1 h/2 to 3 h/4 to 6 h/7 h or more). Intensity of PA was reported as 1) takes it easy without getting out of breath and/or sweaty, 2) gets out of breath and/or sweaty, 3) gets almost exhausted [34]. The questions on frequency of leisure time MVPA and duration, i.e. weekly leisure time MVPA, have been examined for reliability and validity [37]. Intraclass correlation coefficients for reliability were 0.71 for frequency and 0.73 for duration. Validity against VO_2peak_ was acceptable with Spearman correlation coefficients (r_s_) of 0.39 with frequency and 0.33 with duration, but low against total energy expenditure and physical activity level (PAL) measured by accelerometers [37]. We also assessed time spent on TV, video, electronical devices, DVD or PC outside school, with response options 1) less than ½ hour, 2) ½ to 1 h, 3) 2 to 3 h a day [34]. Finally, approximate hours of sleep at night on weekdays was reported (8 h or less/9 h/10 h/11 h/12 h or more) [31].

Mothers’ height in cm was self-reported at baseline. They reported their own weight in kg at the 7-year follow-up and their BMI was calculated (kg/m^2^). Questions regarding the mothers’ exercise were part of the Physical Activity and Pregnancy Questionnaire (PAPQ) [39], previously used in this study [25]. The mothers reported whether they met the recommendation from the Norwegian Directorate of Health valid at the time to perform 30 min of daily PA (yes/no) and if they currently exercised regularly, i.e. performed weekly physical activity to maintain or improve physical fitness (yes/no). If so, they reported the average frequency of exercise per week (1/2/3/4/5 days or more) and average duration of each exercise session with response options 1) 0–30 min, 2) 31–60 min, 3) 61–90 min and 4) > 90 min. Intensity of mother’s PA was reported as 1) a little strenuous, 2) strenuous or 3) very strenuous, and she reported minutes per day spent cycling/walking/running to and from work (none/20–30 min/31–60 min/> 60 min). The PAPQ has shown good correlation with PAL measured by accelerometers for time spent in high intensity activities (r_s_ = 0.59), moderate for time spent standing/moving (r_s_ = 0.36) and fair for sitting/lying (r_s_ = 0.29) [40].

### Baseline variables

At baseline (week 18–22 of pregnancy), the women’s age, height, pre-pregnancy weight, exercise, parity, birth weight of previous children and diabetes in near family (parents, siblings or children) were self-reported. Weight was measured, and pre-pregnancy BMI and baseline BMI was calculated. Socioeconomic status (SES) was calculated based on mother’s education and occupation, according to Hollingshead Two-Factor Index of Social Position [41]. Information about the children’s sex, birth weight, gestational age, length, head circumference, type of delivery and admittance to the neonatal intensive care unit (NICU) were retrieved from medical charts after birth.

### Statistical analyses and power calculation

All analyses were performed using IBM SPSS Statistics 27. Statistical significance was set at two-sided *p* values < 0.05. Group differences were analysed using chi-square statistics for categorical data, Student’s t test for continuous data with a normal distribution and Mann-Whitney U test for ordinal data and continuous data with a non-normal distribution. In correlation analyses we used Pearson correlation coefficient for continuous and normally distributed data and Spearman correlation coefficient for ordinal data. To assess normality, we visually inspected histograms and Q-Q plots of the residuals. In addition to the main analyses, we performed stratified analyses by sex, subgroup analyses including women who adhered to the exercise protocol compared to the control group and sensitivity analyses excluding children born preterm, children who had been admitted to the NICU and children with diseases or health problems at the 7-year follow-up. We analysed the total sample when there were no differences between the intervention and the control group.

In a power calculation prior to the follow-up study, we assumed that approximately 50% of the eligible children (i.e. *n* = 400) would attend the follow-up study. With a power (β) of 80% and significance level (α) of 0.05, we would be able to detect group differences in BMI of 0.4 kg/m^2^ based on a standard deviation (SD) of 1.3 for 7-year-old Norwegian boys and girls [42].

## Results

**Fig. 1:**
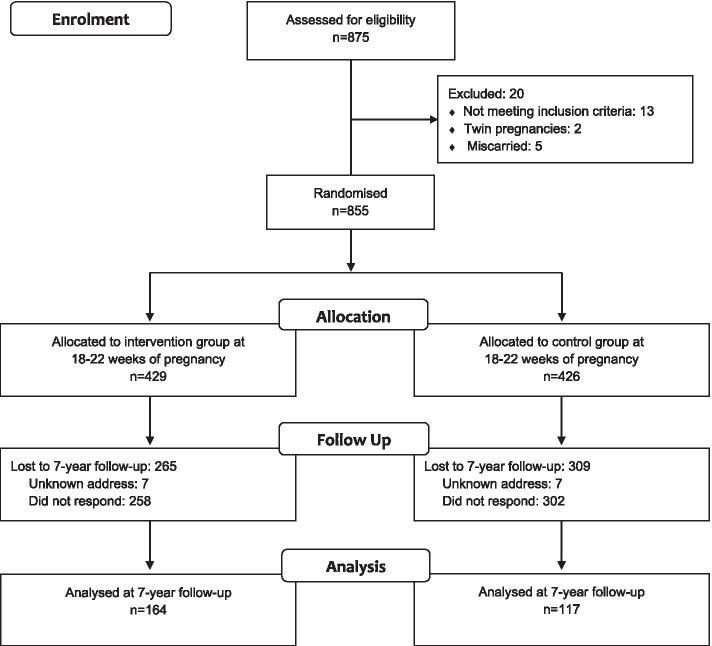
Flow of study participants

Figure 1 shows the flow of participants. At inclusion, 429 women were randomised to the intervention group and 426 women to the control group. At follow-up, seven women in each group could not be reached because of unknown address. A further 258 women in the intervention group and 302 women in the control group did not respond. In total, data on height, weight and PA were provided for 164 children and mothers in the intervention group and 117 children and mothers in the control group (Fig. 1).

Table 1 shows that characteristics of mothers and children were comparable between the two groups, except for a higher proportion of boys in the intervention group than in the control group (*p* = 0.020).

**Table 1:** Characteristics of mothers and children in the intervention and the control group

	Intervention (***n*** = 164)		Control (***n*** = 117)		
	Mean	(SD)	Mean	(SD)	***p*** value
**Maternal characteristics at baseline**					
Age, years	30.5	(3.9)	30.7	(4.2)	0.755
Age > 40 years, n (%)	3	(1.8)	2	(1.7)	1.000
Height, cm	168.8	(5.5)	168.3	(6.1)	0.447
Weight, kg	69.4	(9.3)	69.6	(10.0)	0.863
BMI, kg/m^2^	24.3	(2.8)	24.6	(3.2)	0.522
Pre-pregnancy BMI ≥25 kg/m^2^, n (%)	29	(17.7)	20	(17.1)	0.898
SES	4.0	(0.8)	4.1	(0.8)	0.502
Exercise sessions per week	1.9	(1.4)	1.7	(1.4)	0.160
Diabetes in near family^a^, n (%)	13	(8.3)	13	(12.4)	0.277
Parity, n (%)					
0	96	(58.5)	63	(53.8)	
1	51	(31.1)	38	(32.5)	0.369
2 or more	17	(10.4)	16	(13.7)	
Birth weight of previous child ≥4500 g, n (%)	3	(1.8)	1	(0.9)	0.643
**Child characteristics at birth**					
Gestational age, weeks	39.9	(1.7)	40.1	(1.3)	0.292
Birth weight, g	3477	(608)	3580	(440)	0.102
Length^b^, cm	49.7	(2.8)	50.2	(1.8)	0.154
Head circumference^c^, cm	34.9	(1.8)	35.3	(1.4)	0.047
Male sex, n (%)	96	(58.5)	52	(44.4)	0.020
Older siblings, n (%)	68	(41.5)	54	(46.2)	0.434
Vaginal delivery, n (%)	143	(87.2)	104	(88.9)	0.668
Prematurity, n (%)	7	(4.3)	3	(2.6)	0.530
Admitted to NICU^d^, n (%)	6	(3.7)	1	(0.9)	0.245
**Age at follow-up, years**	7.4	(0.3)	7.4	(0.3)	0.294

*BMI* body mass index, *SES* socioeconomic status (range 1–5, where 5 is highest), *SD* standard deviation, *NICU* neonatal intensive care unit

^a^Missing data for seven mothers in the intervention group and 12 mothers in the control group

^b^Missing data for five children in the intervention group and six children in the control group

^c^Missing data for one child in the intervention group

^d^Missing data for three children in the intervention group and two children in the control group

At baseline, 64 (39.0%) women in the intervention group and 33 (28.2%) women in the control group reported to perform aerobic exercise at moderate intensity three or more times per week before pregnancy. During the intervention period, only 93 (56.7%) women in the intervention group adhered to the exercise protocol. In the control group, 14 (12.0%) women reported exercising as much as requested in the exercise protocol.

### Anthropometry and physical activity of the children

Table 2 shows height, weight, iso-BMI, and PA of the children at 7 years. There were no differences in height or weight, and mean iso-BMI in both groups were 19.4 kg/m^2^. There were no group differences in proportion of children with overweight. In total, 109 (66.5%) children in the intervention group and 68 (59.1%) children in the control group had at least 1 h per day of MVPA. There were no differences in frequency of leisure time MVPA, weekly leisure time MVPA or intensity of PA, daily use of electronical devices or hours of sleep on a weeknight (Table 2). Parents reported that 36 (22.4%) children in the intervention group and 19 (16.8%) children in the control group had a disease or health problem (*p* = 0.259). None had cancer, diabetes, cerebral palsy, mental retardation, Asperger’s syndrome, or chronic fatigue syndrome/myalgic encephalomyelitis.

**Table 2:** Anthropometry and physical activity of children in the intervention and the control group at 7 years of age

	Intervention (***n*** = 164)		Control (***n*** = 117)		
	Mean	(SD)	Mean	(SD)	***p*** value
**Anthropometry**					
Height^a^, cm	127.4	(5.8)	127.0	(5.9)	0.631
Weight^b^, kg	25.2	(4.1)	25.3	(4.8)	0.963
BMI^c^, kg/m^2^	15.5	(1.7)	15.6	(2.6)	0.865
Iso-BMI^c^, kg/m^2^	19.4	(2.7)	19.3	(3.1)	0.957
Iso-BMI ≥25 kg/m^2c^, n (%)	10	(7.6)	7	(7.2)	0.918
	**n**	**(%)**	**n**	**(%)**	
**Daily MVPA**^**d**^					
1 h or more	109	(66.5)	68	(59.1)	0.211
**Frequency of leisure time MVPA**					
Never	1	(0.6)	2	(1.7)	
Once a month or less	4	(2.4)	1	(0.9)	
Once a week	33	(20.1)	33	(28.2)	0.057
2 to 3 times a week	93	(56.7)	67	(57.3)	
4 to 6 times a week	30	(18.3)	12	(10.3)	
Every day	3	(1.8)	2	(1.7)	
**Weekly leisure time MVPA**					
None	4	(2.4)	5	(4.3)	
1 h	51	(31.1)	36	(30.8)	
2 to 3 h	79	(48.2)	59	(50.4)	0.551
4 to 6 h	28	(17.1)	13	(11.1)	
7 h or more	2	(1.2)	4	(3.4)	
**Intensity of PA**^**e**^					
Easy	22	(13.4)	12	(10.3)	
Out of breath	137	(83.5)	98	(84.5)	0.305
Almost exhausted	5	(3.0)	6	(5.2)	
**Daily use of electronical devices**^**f**^					
Less than ½ hour	13	(8.0)	9	(7.7)	
½ to 1 h	118	(72.4)	73	(62.4)	0.091
2 to 3 h	32	(19.6)	35	(29.9)	
**Hours of sleep per weeknight**^**f**^					
8 h or less	3	(1.8)	1	(0.9)	
9 h	13	(8.0)	10	(8.5)	
10 h	92	(56.4)	74	(63.2)	0.375
11 h	53	(32.5)	31	(26.5)	
12 h or more	2	(1.2)	1	(0.9)	

*BMI* body mass index, *Iso-BMI* BMI adjusted for age and sex, *MVPA* moderate to vigorous physical activity, *PA* physical activity, *SD* standard deviation

^a^Missing data for seven children in the intervention group and nine children in the control group

^b^Missing data for 31 children in the intervention group and 20 children in the control group

^c^Missing data for 32 children in the intervention group and 20 children in the control group

^d^Missing data for two children in the control group

^e^Missing data for one child in the control group

^f^Missing data for one child in the intervention group

Stratified by sex (Additional file 1: Tables S1a and S1b), a higher proportion of boys in the control group (*n* = 21; 40.4%) spent 2–3 h a day on electronical devices compared with 18 (18.8%) boys in the intervention group (*p* = 0.030). When we performed subgroup analysis including the children of women who adhered to the protocol (*n* = 93) compared with the control group, iso-BMI and distribution of PA were similar with no significant group differences (data not shown). When we excluded children born preterm (*n* = 10), children admitted to the NICU (*n* = 7) and children with diseases or health problems at the 7-year follow-up (*n* = 55), results were unchanged (data not shown).

### Anthropometry and physical activity of the mothers

There were no significant group differences in the mothers’ height, weight, BMI or exercise at follow-up (Table 3). In all, 114 (69.9%) women in the intervention group, and 90 (76.9%) women in the control group reported at least 30 min of daily PA, and 114 (69.9%) women in the intervention group and 76 (65.0%) women in the control group performed regular exercise.

**Table 3:** Anthropometry and physical activity of mothers in the intervention and the control group at the 7-year follow-up

	Intervention (***n*** = 164)		Control (***n*** = 117)		
	Mean	(SD)	Mean	(SD)	***p*** value
**Anthropometry**					
Height, cm	168.8	(5.5)	168.3	(6.1)	0.447
Weight^a^, kg	65.5	(9.6)	67.1	(11.3)	0.221
BMI^a^, kg/m^2^	22.9	(3.0)	23.7	(3.7)	0.093
	**n**	**(%)**	**n**	**(%)**	
**Daily PA**^**b**^					
30 min or more	114	(69.9)	90	(76.9)	0.195
**Regular exercise**^**b**^					
Yes	114	(69.9)	76	(65.0)	0.379
**Weekly frequency of exercise**^**b**^					
1 day	18	(11.0)	13	(11.1)	
2 days	46	(28.2)	31	(26.5)	
3 days	35	(21.5)	21	(17.9)	0.406
4 days	8	(4.9)	6	(5.1)	
5 or more days	7	(4.3)	5	(4.3)	
N/A	49	(30.1)	41	(35.0)	
**Duration of exercise session**^**b**^					
0–30 min	3	(1.8)	2	(1.7)	
31–60 min	80	(49.1)	60	(51.3)	
61–90 min	30	(18.4)	14	(12.0)	0.165
90 min or more	1	(0.6)	0	(0.0)	
N/A	49	(30.1)	41	(35.0)	
**Intensity of exercise**^**b**^					
A little strenuous	1	(0.6)	2	(1.7)	
Strenuous	63	(38.7)	41	(35.0)	0.396
Very strenuous	50	(30.7)	33	(28.2)	
N/A	49	(30.1)	41	(35.0)	
**Commuting PA**^**b**^					
None	86	(52.8)	52	(44.4)	
20–30 min	45	(27.6)	33	(28.2)	0.111
30–60 min	27	(16.6)	27	(23.1)	
> 60 min	5	(3.1)	5	(4.3)	

*BMI* body mass index, *PA* physical activity, *N/A* not applicable (no regular exercise); Regular exercise = weekly physical activity to maintain or improve physical fitness, *SD* standard deviation

^a^Missing data for nine mothers in the intervention group and five mothers in the control group

^b^Missing data for one mother in the intervention group

When we performed subgroup analysis of women who adhered to the protocol, mothers in the intervention group had a lower mean weight (64.0 kg, SD8.0 vs. 67.1 kg, SD11.3; *p* = 0.026) and BMI (22.6 kg/m^2^, SD2.7 vs. 23.7 kg/m^2^, SD3.7; *p* = 0.019) compared with mothers in the control group.

### Association between children’s and mothers’ body mass index and physical activity

Table 4 shows the correlation between children’s and mothers’ BMI and PA in the total sample. There was an association between children’s iso-BMI and mothers’ BMI (r = 0.254, *p* < 0.001). The children’s weekly leisure time MVPA correlated with the mothers’ daily PA (r_s_ = 0.129, *p* = 0.030) and the children's intensity of PA correlated with mothers’ intensity of exercise (r_s_ = 0.212, *p* = 0.003).

**Table 4:** Correlation coefficients for the association of children’s iso-BMI and PA with mothers’ BMI and PA in the total sample at the 7-year follow-up

Mothers	BMI^e^		Daily PA^f^		Regular exercise^f^		Weekly frequency of exercise^g^		Duration of exercise session^g^		Intensity of exercise^g^		Commuting PA^f^	
Children	r	*p*	r_s_	*p*	r_s_	*p*	r_s_	*p*	r_s_	*p*	r_s_	*p*	r_s_	*p*
Iso-BMI^a^	0.254	0.000												
Daily MVPA^b^	–	–	0.102	0.088	0.054	0.374	0.080	0.272	0.089	0.223	0.126	0.085	0.052	0.387
Frequency of leisure time MVPA	–	–	0.112	0.061	0.060	0.314	0.096	0.187	0.027	0.712	0.100	0.168	0.042	0.487
Weekly leisure time MVPA	–	–	0.129	0.030	0.019	0.747	0.079	0.281	0.076	0.296	0.106	0.146	−0.030	0.621
Intensity of PA^c^	–	–	−0.027	0.658	0.012	0.837	0.030	0.684	0.003	0.964	0.212	0.003	0.060	0.320
Daily use of electronical devices^d^	–	–	−0.043	0.474	−0.062	0.306	−0.001	0.091	0.019	0.797	0.035	0.633	−0.002	0.979

*BMI* body mass index, *Iso-BMI* BMI adjusted for age and sex, *MVPA* moderate to vigorous physical activity, *PA* physical activity; Regular exercise = weekly physical activity to maintain or improve physical fitness, *r* Pearson correlation coefficient, *r*_*s*_ Spearman correlation coefficient

^a^Missing data for 32 children in the intervention group and 20 children in the control group

^b^Missing data for two children in the control group

^c^Missing data for one child in the control group

^d^Missing data for one child in the intervention group

^e^Missing data for nine mothers in the intervention group and five mothers in the control group

^f^Missing data for one mother in the intervention group

^g^Answers requested only for mothers reporting regular exercise (*n* = 114 in the intervention group; *n* = 76 in the control group)

Stratified by group, children’s iso-BMI correlated with mothers’ BMI in both groups. In the intervention group, both daily MVPA and PA intensity of the children correlated with mothers’ intensity of exercise (Additional file 2: Table S2a). In the control group, both frequency of leisure time MVPA and PA intensity of the children correlated with mothers’ intensity of exercise (Additional file 2: Table S2b).

### Loss to follow-up

There was a lower proportion of non-participants in the intervention group (*n* = 265; 61.8%) than in the control group (*n* = 309; 72.5%) (*p* < 0.001). There were no differences between participants and non-participants in either group regarding maternal age, age > 40 years, height, weight, pre-pregnancy or baseline BMI, parity, birth weight of previous baby or diabetes in near family at baseline or children’s gestational age, birth weight, length, head circumference, sex, mode of delivery, proportion of preterm birth or older siblings (data not shown). In the intervention group, mean exercise sessions per week before pregnancy was slightly higher among mothers of participating children compared with non-participants (1.8, SD1.4 vs. 1.7, SD1.4, *p* = 0.048). In the control group, mean SES was 4.0 (SD0.8) among mothers of participating children compared with 3.8 (SD1.0) among non-participants (*p* = 0.004). Further, only one (0.9%) participating control child had been admitted to the NICU compared with 18 (5.9%) non-participating control children (*p* = 0.026).

## Discussion

### Main findings

This RCT follow-up study reports on anthropometry and PA of 7-year-old children whose mothers were randomised to moderate intensity exercise at least three times per week during pregnancy or standard antenatal care. As hypothesised, there were no group differences in BMI or PA of the children. Stratified analyses by sex, subgroup analyses including only children of women who adhered to the exercise protocol and sensitivity analyses excluding children born preterm, children admitted to the NICU and children with diseases or health problems at follow-up, gave essentially the same results. We found that children’s iso-BMI correlated with mothers’ BMI. Further, there was an association between the children's leisure time MVPA and intensity of PA with the mothers' daily PA and intensity of exercise.

### Strengths and limitations

A strength of the RCT was the large number of participants and the computerised randomisation procedure used to allocate the women. This entails that misclassification is unlikely to have affected the results. The included women exercised regularly and had baseline BMI within the normal range, indicating they were active and healthy women. Their baseline characteristics were comparable to the large Norwegian Mother and Child Cohort Study [25, 43], indicating a representative selection of Norwegian pregnant women. However, results should be interpreted with caution in pregnant populations with higher BMI, less physically active women and in ethnically diverse populations.

The present follow-up study was based on parent-report. Data was collected electronically using a software, which is a feasible, efficient and low-cost way of collecting data for larger groups of people, ensuring good data quality, and reducing the risk of data entry errors [44]. However, respondents might interpret questions differently or answers could be exaggerated, misremembered, or affected by social desirability bias. We cannot exclude the possibility that this, or other factors such as community or culture, may have influenced the results. Nevertheless, it is unlikely to have affected the groups differently. We do not know whether the parents estimated or measured the child’s weight and height. On an individual level, a Belgium study reported less accuracy when parents estimated their child’s weight and height, and accordingly BMI, than when measurements were performed at home by the parents of children aged 3–7 years [45]. However, on a group level there was no important differences between estimated and measured parent-reports [45]. Moreover, the BMI of the children in our study were similar to parent-reported values in the large national MoBa study with a mean BMI in 7-year old girls (*n* = 1839) and boys (*n* = 1932) of 15.9 kg/m^2^ (SD2.0 and 1.8, respectively) [46]. The PA questions on frequency and duration have shown acceptable reliability and validity [37], and all questions are in line with questions used in other Norwegian and international studies [31, 34–36, 38]. The PAPQ is considered an acceptable method for assessing habitual physical activity and exercise among women at group level [40]. In a priori power calculations we assumed that approximately 50% of the eligible children would attend the follow-up study. Thus, the low follow-up rate of 33%, limits our power to demonstrate differences and correlations, and non-significant findings should therefore be interpreted with caution. However, the mean values were highly similar between the groups and the non-significant correlations generally low, indicating that type II errors were less likely. Furthermore, the low follow-up rate may threaten the representativeness and limit the generalisability of results. A higher proportion of children in the intervention than in the control group attended the follow-up study, however, there were few baseline differences between participants and non-participants. Thus, we can assume that our results are representative for the sample initially included in this study.

### Interpretation

Our study is one of few long-term follow-up studies of exercise during pregnancy. A small RCT of home-based stationary cycling or regular activity during pregnancy of 84 sedentary women reported no differences in height, weight, or BMI of the children at 1 or 7 years. However, children in the intervention group had increased body fat at 7 years, measured by dual-energy x-ray absorptiometry [21]. In three different case-control studies of 65 [18], 40 [19], and 104 [20] exercising women, children of women who continued to exercise in pregnancy weighed less and had less body fat than control children at birth [18] and at 5 years of age [19], suggesting that exercise during pregnancy reduced subcutaneous fat mass of the offspring. However, at 1 year, all anthropometric measurements were similar [20]. Limitations with these studies include several potential confounders and highly selected and relatively small groups of healthy, exercising women. A large population-based study of 5125 Greek children reported that retrospectively recalled PA during pregnancy were significantly associated with obesity in the offspring at 8 years of age [47]. However, this study included a large proportion of pre-pregnancy overweight and obese women, not necessarily representative for a healthy pregnant population. This may indicate that preconceptual health is more important than prenatal exercise or that other mechanisms of childhood obesity play a role. In the present RCT follow-up study, we found no evidence that children of mothers receiving regular exercise during pregnancy had a more unfavourable body composition than children of mothers receiving standard care. Consistent with our results, a meta-analysis of 135 studies (*n* = 166,094 women) from 32 countries revealed no association between prenatal exercise and neonatal outcomes or body composition in terms of body fat percentage, body weight and BMI in childhood [48]. Further, data from the large Danish National Birth Cohort Study, including 40,280 mother-child pairs, supports our findings that maternal exercise during pregnancy are not related to children’s BMI or risk of overweight [49].

Not meeting the recommendation of 1 h daily MVPA [6] is associated with an increased chance of overweight and obesity [50]. Parents reported that 55.6–60.3% of the girls and 63.5–70.8% of the boys in the two groups, respectively, met this recommendation. These proportions seem lower than what has been found by use of accelerometer measurements of PA in Norwegian children, where 87% of girls and 94% of boys met the recommendation at 6 years of age, but the proportions fell to 64 and 81% at 9 years of age [51]. In stratified analyses by sex, boys in the control group spent significantly more time on electronical devices than boys in the intervention group. This is probably a random finding. Even though longer screen viewing time in children has been associated with more sedentary behaviour, which might displace PA during early childhood [52], we have no reason to believe that it affected the children’s PA in this study. We found that daily MVPA, frequency of leisure time MVPA, weekly leisure time MVPA and intensity of PA were similar in the two groups.

The present results also showed that most women in both groups performed daily PA and exercised regularly 7 years after the intervention period. When we performed subgroup analyses of women adhering to the exercise protocol, their weight and BMI were lower compared with the control group, consistent with other studies suggesting that an exercise intervention during pregnancy has a positive effect on mothers in the long term [21, 53–55]. However, the subgroup analysis did not change the children’s results.

The associations between children’s and mothers’ BMI and PA both in the total material and in each group, suggest that mother’s adherence to a healthy lifestyle could influence her child’s health. These findings are supported by a British study, reporting a direct association between PA levels measured by accelerometery in 554 mothers and their 4-year-old children [56]. Furthermore, a healthy lifestyle including regular exercise and a healthy BMI of the mother during their offspring’s childhood and adolescence has been associated with a substantially reduced risk of obesity in the children [22].

Our results add to the existing knowledge on exercise during pregnancy. Both in previous [10, 11] and the current follow-up study, we have shown that exercise during pregnancy does not adversely affect childhood outcomes. As pregnancy may be a time when the level of PA declines [43, 57], interventions for encouraging pregnant women to be physically active seem beneficial, given the adverse health effects of inactivity, overweight and excessive gestational weight gain [16, 43, 58–60]. Thus, promoting a healthy lifestyle, both before, during and after pregnancy, may prevent chronic disease risk in more than one generation.

## Conclusions

In this RCT follow-up study, we have shown that randomisation to regular moderate intensity exercise during pregnancy did not affect children’s BMI or PA at 7 years of age. Furthermore, we found that children’s and mothers’ BMI and PA at the 7-year follow-up were correlated. As women participating in this study were initially healthy, our results indicate that good prenatal and postpartum maternal health should be encouraged as it may influence the health of the next generation.

## Supplementary Information

**Additional file 1: Table S1a.** Anthropometry and physical activity of girls in the intervention and the control group at 7 years of age. **Table S1b.** Anthropometry and physical activity of boys in the intervention and the control group at 7 years of age.

**Additional file 2: Table S2a.** Correlation coefficients for the association of children’s iso-BMI and PA with mothers’ BMI and PA in the intervention group at the 7-year follow-up. **Table S2b.** Correlation coefficients for the association of children’s iso-BMI and PA with mothers’ BMI and PA in the control group at the 7-year follow-up.

## Data Availability

The datasets generated and/or analysed during the current study are not publicly available due to permission has not been applied for from neither the participants nor the Ethical Committee but are available from the corresponding author on reasonable request.

## Abbreviations

BMI: Body Mass Index
MVPA: Moderate to vigorous physical activity
NICU: Neonatal intensive care unit
PA: Physical activity
PAL: Physical activity level
PAPQ: Physical Activity and Pregnancy Questionnaire
RCT: Randomised controlled trial
SES: Socioeconomic status
