# Supplementary material for: Body mass index and physical activity in seven-year-old children whose mothers exercised during pregnancy: follow-up of a multicentre randomised controlled trial
Source: BMC Pediatr. 2021 Nov 8;21:496. doi: 10.1186/s12887-021-02952-1 (PMC8574029; doi:10.1186/s12887-021-02952-1)
Supplement: Supplementary file 1 — Additional file 1: Table S1a. Anthropometry and physical activity of girls in the intervention and the control group at 7 years of age. Table S1b. Anthropometry and physical activity of boys in the intervention and the control group at 7 years of age. [file 12887_2021_2952_MOESM1_ESM.pdf]

Table S1a. Anthropometry and physical activity of girls in the intervention and the control group at 7 years of age.

Girls	Intervention (n = 68)		Control (n = 65)		
	**Mean**	**(SD)**	**Mean**	**(SD)**	***p* value**
**Anthropometry**					
Height[a], cm	126.6	(5.2)	126.4	(4.9)	0.832
Weight[b], kg	24.9	(3.3)	25.0	(5.3)	0.917
BMI[c], kg/m^2	15.5	(1.4)	15.5	(3.3)	0.975
Iso-BMI[c], kg/m^2	19.4	(2.5)	19.4	(3.5)	0.881
Iso-BMI ≥25 kg/m^2[c], n (%)	6	(11.1)	4	(7.8)	0.742
	**n**	**(%)**	**n**	**(%)**	
**Daily amount of MVPA[d]**					
1 hour or more	41	(60.3)	35	(55.6)	0.583
**Frequency of leisure time MVPA**					
Never	0	(0.0)	0	(0.0)	
Once a month or less	3	(4.4)	0	(0.0)	
Once a week	16	(23.5)	22	(33.8)	0.491
2 to 3 times a week	38	(55.9)	35	(53.8)	
4 to 6 times a week	10	(14.7)	7	(10.8)	
Every day	1	(1.5)	1	(1.5)	
**Weekly leisure time MVPA**					
None	4	(5.9)	1	(1.5)	
1 hour	19	(27.9)	24	(36.9)	
2 to 3 hours	36	(52.9)	31	(47.7)	0.858
4 to 6 hours	9	(13.2)	8	(12.3)	
7 hours or more	0	(0.0)	1	(1.5)	
**Intensity of PA**					
Easy	12	(17.6)	6	(9.2)	
Out of breath	56	(82.4)	57	(87.7)	0.099
Almost exhausted	0	(0.0)	2	(3.1)	
**Daily use of electronical devices[e]**					
Less than ½ hour	6	(9.0)	4	(6.2)	
½ to 1 hour	47	(70.1)	47	(72.3)	0.753
2 to 3 hours	14	(20.9)	14	(21.5)	
**Hours of sleep per weeknight[e]**					
8 hours or less	2	(3.0)	1	(1.5)	
9 hours	5	(7.5)	4	(6.2)	
10 hours	41	(61.2)	44	(67.7)	0.841
11 hours	18	(26.9)	16	(24.6)	
12 hours or more	1	(1.5)	0	(0.0)	

BMI = body mass index; Iso-BMI = BMI adjusted for age and sex; MVPA = moderate to vigorous physical activity; PA = physical activity; SD = standard deviation

[a]Missing data for four girls in the intervention group and five girls in the control group

[b]Missing data for 13 girls in the intervention group and 14 girls in the control group

[c]Missing data for 14 girls in the intervention group and 14 girls in the control group

[d]Missing data for two girls in the control group

[e]Missing data for one girl in the intervention group

Table S1b. Anthropometry and physical activity of boys in the intervention and the control group at 7 years of age.

Boys	Intervention (n = 96)		Control (n = 52)		
	**Mean**	**(SD)**	**Mean**	**(SD)**	***p* value**
**Anthropometry**					
Height[a], cm	127.9	(6.1)	127.8	(6.9)	0.929
Weight[b], kg	25.5	(4.5)	25.6	(4.3)	0.904
BMI[b], kg/m^2	15.5	(1.9)	15.6	(1.6)	0.790
Iso-BMI[b], kg/m^2	19.3	(2.9)	19.3	(2.6)	0.965
Iso-BMI ≥25 kg/m^2[b], n (%)	4	(5.1)	3	(6.5)	0.710
	**n**	**(%)**	**n**	**(%)**	
**Daily amount of MVPA**					
1 hour or more	68	(70.8)	33	(63.5)	0.358
**Frequency of MVPA**					
Never	1	(1.0)	2	(3.8)	
Once a month or less	1	(1.0)	1	(1.9)	
Once a week	17	(17.7)	11	(21.2)	0.089
2 to 3 times a week	55	(57.3)	32	(61.5)	
4 to 6 times a week	20	(20.8)	5	(9.6)	
Every day	2	(2.1)	1	(1.9)	
**Weekly amount of MVPA**					
None	0	(0.0)	4	(7.7)	
1 hour	32	(33.3)	12	(23.1)	
2 to 3 hours	43	(44.8)	28	(53.8)	0.673
4 to 6 hours	19	(19.8)	5	(9.6)	
7 hours or more	2	(2.1)	3	(5.8)	
**Intensity of PA[c]**					
Easy	10	(10.4)	6	(11.8)	
Out of breath	81	(84.4)	41	(80.4)	0.982
Almost exhausted	5	(5.2)	4	(7.8)	
**Daily use of electronical devices**					
Less than ½ hour	7	(7.3)	5	(9.6)	
½ to 1 hour	71	(74.0)	26	(50.0)	0.030
2 to 3 hours	18	(18.8)	21	(40.4)	
**Amount of sleep per weeknight**					
8 hours or less	1	(1.0)	0	(0.0)	
9 hours	8	(8.3)	6	(11.5)	
10 hours	51	(53.1)	30	(57.7)	0.441
11 hours	35	(36.5)	15	(28.8)	
12 hours or more	1	(1.0)	1	(1.9)	

BMI = body mass index; Iso-BMI = BMI adjusted for age and sex; MVPA = moderate to vigorous physical activity; PA = physical activity; SD = standard deviation

[a]Missing data for three boys in the intervention group and four boys in the control group

[b]Missing data for 18 boys in the intervention group and six boys in the control group

[c]Missing data for one boy in the control group
