# Supplementary material for: Body mass index and physical activity in seven-year-old children whose mothers exercised during pregnancy: follow-up of a multicentre randomised controlled trial
Source: BMC Pediatr. 2021 Nov 8;21:496. doi: 10.1186/s12887-021-02952-1 (PMC8574029; doi:10.1186/s12887-021-02952-1)
Supplement: Supplementary file 2 — Additional file 2: Table S2a. Correlation coefficients for the association of children’s iso-BMI and PA with mothers’ BMI and PA in the intervention group at the 7-year follow-up. Table S2b. Correlation coefficients for the association of children’s iso-BMI and PA with mothers’ BMI and PA in the control group at the 7-year follow-up. [file 12887_2021_2952_MOESM2_ESM.pdf]

Table S2a. Correlation coefficients for the association of children's iso-BMI and PA with mothers' BMI and PA in the intervention group at the 7-year follow-up.

Mothers	BMI[c]		Daily PA[d]		Regular exercise[d]		Weekly frequency of exercise[e]		Duration of exercise session[e]		Intensity of exercise[e]		Commuting PA[d]	
**Children**	**r**	***p***	**r_s**	***p***	**r_s**	***p***	**r_s**	***p***	**r_s**	***p***	**r_s**	***p***	**r_s**	***p***
Iso-BMI[a]	0.208	0.019												
Daily MVPA	-	-	0.126	0.108	0.070	0.376	0.075	0.430	0.036	0.701	0.232	0.013	0.073	0.353
Frequency of leisure time MVPA	-	-	0.105	0.181	0.014	0.855	0.014	0.882	-0.066	0.482	0.014	0.886	0.042	0.597
Weekly leisure time MVPA	-	-	0.117	0.137	-0.060	0.450	0.021	0.822	0.003	0.974	0.091	0.338	-0.045	0.572
Intensity of PA	-	-	-0.075	0.339	-0.006	0.942	0.062	0.513	-0.004	0.969	0.204	0.029	0.088	0.266
Daily use of electronical devices[b]	-	-	-0.038	0.632	-0.137	0.083	0.008	0.933	-0.030	0.755	0.048	0.612	-0.004	0.964

BMI = body mass index; Iso-BMI = BMI adjusted for age and sex; MVPA = moderate to vigorous physical activity; PA = physical activity; Regular exercise = weekly physical activity to maintain or improve physical fitness; r = Pearson correlation coefficient; r_s = Spearman correlation coefficient

[a]Missing data for 32 children in the intervention group

[b]Missing data for one child in the intervention group

[c]Missing data for nine mothers in the intervention group

[d]Missing data for one mother in the intervention group

[e]Answers requested only for mothers reporting regular exercise (n=114 in the intervention group)

Table S2b. Correlation coefficients for the association of children's iso-BMI and PA with mothers' BMI and PA in the control group at the 7-year follow-up.

**Mothers**	**BMI**[d]		**Daily PA**		**Regular exercise**		**Weekly frequency of exercise**		**Duration of exercise session**		**Intensity of exercise**		**Commuting PA**	
**Children**	**r**	***p***	$\mathbf{r_s}$	***p***	$\mathbf{r_s}$	***p***	$\mathbf{r_s}$	***p***	$\mathbf{r_s}$	***p***	$\mathbf{r_s}$	***p***	$\mathbf{r_s}$	***p***
Iso-BMI[a]	0.304	0.003												
Daily MVPA[c]	-	-	0.082	0.384	0.024	0.797	0.087	0.460	0.156	0.180	-0.026	0.825	0.039	0.682
Frequency of leisure time MVPA	-	-	0.143	0.125	0.110	0.237	0.214	0.063	0.184	0.112	0.234	0.042	0.067	0.475
Weekly leisure time MVPA	-	-	0.154	0.097	0.121	0.194	0.168	0.147	0.213	0.065	0.133	0.252	0.002	0.979
Intensity of PA[b]	-	-	0.034	0.714	0.044	0.640	-0.016	0.888	0.040	0.734	0.230	0.046	0.006	0.945
Daily use of electronical devices	-	-	-0.066	0.479	0.038	0.680	-0.010	0.929	0.125	0.283	0.023	0.844	-0.021	0.823

BMI = body mass index; Iso-BMI = BMI adjusted for age and sex; MVPA = moderate to vigorous physical activity; PA = physical activity; Regular exercise = weekly physical activity to maintain or improve physical fitness; r = Pearson correlation coefficient; r_s = Spearman correlation coefficient

[a]Missing data for children in the control group

[b]Missing data for one child in the control group

[c]Missing data for two children in the control group

[d]Missing data for five mothers in the control group

[e]Answers requested only for mothers reporting regular exercise (n=76 in the control group)
